# Consequences of splitting whole-genome sequencing effort over multiple breeds on imputation accuracy

**DOI:** 10.1186/s12863-014-0105-8

**Published:** 2014-10-03

**Authors:** Aniek C Bouwman, Roel F Veerkamp

**Affiliations:** Animal Breeding and Genomics Centre, Wageningen UR Livestock Research, P.O. Box 338, 6700 AH Wageningen, Netherlands

**Keywords:** Imputation, Multi-breed, Next generation sequencing

## Abstract

**Background:**

The aim of this study was to determine the consequences of splitting sequencing effort over multiple breeds for imputation accuracy from a high-density SNP chip towards whole-genome sequence. Such information would assist for instance numerical smaller cattle breeds, but also pig and chicken breeders, who have to choose wisely how to spend their sequencing efforts over all the breeds or lines they evaluate. Sequence data from cattle breeds was used, because there are currently relatively many individuals from several breeds sequenced within the 1,000 Bull Genomes project. The advantage of whole-genome sequence data is that it carries the causal mutations, but the question is whether it is possible to impute the causal variants accurately. This study therefore focussed on imputation accuracy of variants with low minor allele frequency and breed specific variants.

**Results:**

Imputation accuracy was assessed for chromosome 1 and 29 as the correlation between observed and imputed genotypes. For chromosome 1, the average imputation accuracy was 0.70 with a reference population of 20 Holstein, and increased to 0.83 when the reference population was increased by including 3 other dairy breeds with 20 animals each. When the same amount of animals from the Holstein breed were added the accuracy improved to 0.88, while adding the 3 other breeds to the reference population of 80 Holstein improved the average imputation accuracy marginally to 0.89. For chromosome 29, the average imputation accuracy was lower. Some variants benefitted from the inclusion of other breeds in the reference population, initially determined by the MAF of the variant in each breed, but even Holstein specific variants did gain imputation accuracy from the multi-breed reference population.

**Conclusions:**

This study shows that splitting sequencing effort over multiple breeds and combining the reference populations is a good strategy for imputation from high-density SNP panels towards whole-genome sequence when reference populations are small and sequencing effort is limiting. When sequencing effort is limiting and interest lays in multiple breeds or lines this provides imputation of each breed.

## Background

Next generation sequencing techniques have developed very rapidly over the last decade resulting in an increase in the number of sequenced individuals. Even though whole-genome sequencing costs are reducing, sequencing large populations is financially unfeasible. When genotyping large animal populations for high-density SNP panels was financially unfeasible, standard practise became that a strategic part of the population was genotyped at a higher density, while the other part of the population was genotyped at a lower density and their low density genotypes were imputed to the higher density to facilitate genomic selection [1-3]. A similar imputation strategy might be used to facilitate the widespread use of whole-genome sequence information in animal breeding. However, the success of imputation towards whole-genome sequence depends on many factors such as size of the reference population, the number of SNP genotyped, the linkage disequilibrium (LD) between typed and to impute variants, the relationships between reference population and individuals to impute, and the sequencing depth [4,5].

Initial whole-genome sequenced reference populations for imputation will be small (less than a hundred animals per breed) since whole-genome sequencing is upcoming and still expensive. Therefore an attractive option might be to combine sequenced individuals from different breeds (or lines) in a reference population to increase the reference population for imputation to whole-genome sequence. In addition to the increase in reference population, it could be hypothesized that for some variants with a low minor allele frequency (MAF), haplotypes in other breeds might aid imputation when they have a higher frequency in those breeds.

Imputation studies using SNP panels usually focus on imputation within a breed. The few studies that included individuals from other breeds in the reference population increased imputation accuracy marginally, but appeared to be successful when the reference population of the breed of interest was small [6] and when the other breeds used had similar genetic background [7-10]. Imputation accuracy improved little when the actual reference population was already sufficiently large for imputation [6,11] and even declined when other breeds were too different [9]. In all studies investigating the benefit of multi-breed reference populations, the information from other breeds were added to the reference population, but in none of the studies the replacement of individuals by other breeds was evaluated. The latter scenario is more likely if a decision needs te be made on which animals from which breed (or line) to sequence. Also, little insight exist in the accuracy of imputation of variants with low MAF and breed specific variants. The advantage of whole-genome sequence data is that it carries the causal mutations, but the questions is whether it is possible to impute the causal variants accurately. A part of the genetic variation observed in traits cannot be captured by 50 K or 777 K SNP chips, this is likely due to causal variants with a very low MAF or even rare alleles. It is therefore important to know how accurate such potential causal variants can be imputed.

The aim of this study was to determine the consequences of splitting sequencing effort over multiple breeds for imputation accuracy from a high-density SNP chip towards whole-genome sequence, and investigate imputation accuracy of variants with low MAF and breed specific variants. To study this we assumed a budget to sequence 80 individuals and interest in 4 breeds selected for the same purpose (i.e. dairy). Such scenario gives 3 options: 1) split sequencing effort over the 4 breeds and perform within breed imputation with a limited reference population; 2) split sequencing effort over the 4 breeds and perform imputation with a multi-breed reference population; or 3) focus sequencing effort on 1 breed only to get a decent size reference population for that breed, but ignoring the other 3 breeds. Such information would assist for instance numerical smaller cattle breeds, and pig and chicken breeding organisations who have to choose wisely how to spend their sequencing efforts over all the breeds or lines they evaluate. However, sequence data from cattle breeds was used, because there are currently relatively many individuals from several breeds sequenced within the 1,000 Bull Genomes project.

## Methods

### Whole-genome sequence data

Whole-genome sequence data were provided by the 1,000 Bull Genomes project (Run 3). Alignment, variant calling, and quality controls were done in a multi-breed population of 429 sequenced key ancestors from 15 different breeds as described by Daetwyler et al. [12]. Genotype calls were improved with BEAGLE using genotype likelihoods from SAMtools and inferred haplotypes in the samples [12], the allele calls from the output of this step were used and assumed to be true genotypes. The Brown Swiss (BSW; n = 43), black and white Holstein (HOL; n = 114), Jersey (JER; n = 27) and Nordic Red Dairy Cattle (Swedish Red and Finnish Ayrshire; RDC; n = 33) bulls were used in this study. Of the 114 black and white Holstein bulls, 14 bulls with lowest or unknown coverage were deleted to end up with 100 Holsteins for the scenarios described below, each with an average coverage of at least 5 fold sequencing depth, with a max of 38 fold sequencing depth. From the other breeds 20 bulls were selected at random from all available bulls for each cross-validation as described below. The bulls from these breeds had an average coverage ranging between 5 and 30 fold sequencing depth, but for 23 BSW bulls the coverage was unknown.

### Scenarios

**Figure 1:**
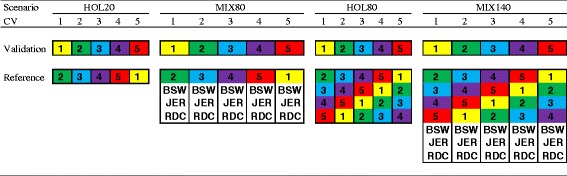
**Cross-validation (CV) scheme for each scenario where each block represents a group of 20 animals.** The 100 Holstein individuals were divided in 5 groups of 20 animals each, and used as validation set once in each scenario. In the reference population the numbered blocks 1 to 5 represent the same 5 groups of 20 Holstein animals as in the validation sets; BSW were groups of 20 Brown Swiss animals; JER were groups of 20 Jersey animals; RDC were groups of 20 Nordic Red Dairy Cattle.

Four scenarios were evaluated to assess the imputation accuracy using different sequenced reference populations to infer genotypes of 20 Holstein validation animals (Figure 1). In the first scenario the 20 Holstein validation animals were imputed with a reference population of 20 sequenced animals from a single breed only, i.e. Holstein (HOL20). In the second scenario the 20 Holstein validation animals were imputed with a reference population of 80 sequenced animals from a mix of dairy breeds, i.e. BSW, HOL, JER, RDC, with 20 animals of each breed (MIX80). In the third scenario the 20 Holstein validation animals were imputed with a single breed reference population of 80 sequenced Holstein animals (HOL80), equal to the number of animals in the MIX80 scenario. The fourth scenario was added to see if there is benefit from other breeds when the initial within-breed reference population is already relatively large. For this scenario the 20 Holstein validation animals were imputed with a multi-breed reference population of 140 animals: 80 HOL, 20 BSW, 20 JER and 20 RDC (MIX140).

### Imputation

Imputation from 777 K SNP chip to whole-genome sequence was undertaken on chromosome 1 (largest chromosome) and 29 (smallest chromosome) using BEAGLE 3.3.2 software [13]. BEAGLE is a population based imputation program, and is widely used because it tends to be relatively fast, especially using whole-genome sequence data [14], and is consistently among the most accurate imputation programs available [15]. BEAGLE was used with default parameter settings assuming unphased genotypes and unrelated individuals.

A five-fold cross-validation was performed for each scenario to assess imputation accuracy (Figure 1). Holstein individuals were randomly divided in five groups of 20 bulls and each group was used as validation set once. In scenario HOL80 all four additional groups were used as reference population (e.g. group 1 was the validation set and group 2, 3, 4, and 5 were the reference set). In HOL20 and MIX80 only the 20 individuals from one of those four groups were used in the reference population (e.g. group 1 was the validation set and group 2 was included in the reference set). For each of the other breeds in MIX80, 20 individuals of each breed were chosen at random from all available animals of that breed, which was repeated for each of the five cross-validations. Similarly, the other breeds were added to the HOL80 reference population for the MIX140 scenario.

The whole-genome sequence data used consisted of di-allelic variants, with the alleles coded as 1 and 2. For validation individuals the genotypes of SNP on Illumina BovineHD BeadChip (Illumina Inc., San Diego, CA; 777,962 SNP) were kept, whereas other variants discovered in the sequence were set to missing.

There are several ways to assess correctness of imputation, but the correlation between observed genotypes and imputed genotype dosages seems to be the most appropriate because it is independent of the MAF [15,16]. Per variant the imputation accuracy (r) was calculated as the correlation between observed and imputed genotype dosages over all five validation groups (i.e. over 100 Holstein). Variants for which either the observed genotypes or the imputed genotypes, or both, were monomorphic in at least one of the five cross-validations were removed. The imputation accuracy ranged between -1 (opposite genotype imputed) and +1 (correct genotype imputed).

Chromosome 1 contained 1,912,451 variants: 1,805,537 SNP and 106,914 short insertions and deletions (indels) according to Run 3 of the 1,000 Bull Genomes project. Of these variants 1,184,875 were segregating in the 100 Holsteins studied of which 38,694 were located on the 777 K SNP chip and thus assumed to be genotyped, leaving 1,146,181 variants to impute (1,069,830 SNP and 76,351 indels). Of these variants to impute 182,964 were Holstein specific (175,227 SNP and 7,737 indels). Holstein specific variants were defined as variants that were segregating at least once in the 100 Holstein but not in any of the individuals of the other three breeds used in this study.

Chromosome 29 contained 670,773 variants: 635,009 SNP and 35,764 short insertions and deletions (indels) according to Run 3 of the 1,000 Bull Genomes project. Of these variants 444,582 were segregating in the 100 Holsteins studied of which 12,865 were located on the 777 K SNP chip and thus assumed to be genotyped, leaving 431,717 variants to impute (405,507 SNP and 26,210 indels). Of these variants to impute 60,202 were Holstein specific (57,858 SNP and 2,344 indels).

### Persistency of phase

Persistency of phase was calculated between the Holstein and each of the other breeds used in the multi-breed (MIX) scenarios, i.e. between 100 HOL and 43 BSW, between 100 HOL and 27 JER, and between 100 HOL and 33 RDC. First, linkage disequilibrium was measured as the correlation coefficient between pairs of loci within a breed (r; here termed *r*_*LD*_). This was calculated within each breed for variants at a certain distance from each other as ${r}_{LD}=\left({p}_{A1B1}{p}_{A2B2}-{p}_{A1B2}{p}_{A2B1}\right)/\sqrt{p_{A1}{p}_{A2}{p}_B{p}_{B2}}$_,_ where *p*_*A*1*B*1_ is the frequency of haplotypes with allele 1 at variant locus A and allele 1 at variant locus B and *p*_*A*1_ is the frequency of allele 1 at variant locus A [17]. Alleles of the variants were numbered consistently over breeds as variants were called over all individuals in Run 3 of the 1,000 Bull Genomes project simultaneously. Second, persistency of phase between two breeds was calculated as the correlation of *r*_*LD*_ (corr(*r*_*LD*_)) of two breeds for a number of variants at a certain distance [18]. Given the large number of sequence variants, this was limited to chromosome 1 and applied to subsets of variants at a distance of 0-1 kb, 5-6 kb, 10-11 kb, 50-51 kb, 100-101 kb, 200-201 kb, and 400-401 kb of each other.

## Results

### Imputation accuracy

For chromosome 1 and 29 the Holstein individuals were imputed from high-density (777 K SNP chip) to whole-genome sequence using a five-fold cross-validation scheme and four different reference populations: HOL20, MIX80, HOL80, and MIX140.

Per cross-validation (i.e. 20 validation animals) the imputation of chromosome 1 took on average 1 h:53 m for HOL20, 7 h:28 m for HOL80, 8 h:40 m for MIX80, and 14 h:13 m for MIX140 on a Unix cluster with Six-Core AMD Opteron^tm^ 8431 processors. The imputation of chromosome 29 took on average 0 h:41 m for HOL20, 2 h:43 m for HOL80, 3 h:0 m for MIX80, and 5 h:41 m for MIX140 per cross-validation on the same Unix cluster.

**Figure 2:**
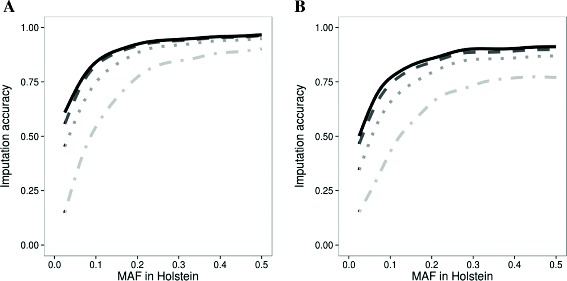
**Imputation accuracy of variants plotted against the minor allele frequency.** Imputation accuracy of variants on chromosome 1 **(A)** and chromosome 29 **(B)** for HOL20 (dotdash line), MIX80 (dotted line), HOL80 (dashed line), and MIX140 (solid line) plotted against the minor allele frequency (MAF) in Holstein. The lines were fitted with a generalized additive model with integrated smoothness estimation using the imputation accuracy over all 5 cross-validations.

For chromosome 1, the average imputation accuracy was 0.70 with a reference population of 20 Holstein (HOL20; Table 1). The addition of 20 BSW, 20 JER, and 20 RDC bulls increased the imputation accuracy to 0.83 (MIX80; Table 1), adding the same amount of animals from the Holstein breed improved the accuracy even up to 0.88 (HOL80; Table 1), and adding 20 BSW, 20 JER, and 20 RDC bulls to a reference population of 80 HOL improved the average imputation accuracy marginally to 0.89 (MIX140; Table 1). For chromosome 29, the average imputation accuracy was lower (Figure 2): 0.59 for HOL20, 0.74 for MIX80, 0.80 for HOL80, and 0.82 for MIX140 (Table 1). Variants with lower MAF had a lower imputation accuracy (Figure 2). With a small reference population of 20 individuals the imputation accuracy increased less with increasing MAF and reached the plateau at a higher MAF compared to the reference populations with 80 or 140 individuals.

**Table 1:** **Average imputation accuracy from the bovine 777 K SNP chip to whole**-**genome sequence on chromosome 1 and 29**

	**Chromosome 1**				**Chromosome 29**			
	**HOL20**	**MIX80**	**HOL80**	**MIX140**	**HOL20**	**MIX80**	**HOL80**	**MIX140**
Sequence (n)	1,912,451	1,912,451	1,91,2451	1,912,451	670,773	670,773	670,773	670,773
777 K chip (n)	41,868	41,868	41,868	41,868	13,556	13,556	13,556	13,556
No variation in reference set (n)^1^	1,178,683	710,480	894,633	616,808	385,137	221,137	283,516	187,904
No variation observed in validation set (n)^1^	−^2^	468,203	284,050	561,875	−^2^	164,000	101,621	197,233
No variation imputed in validation set (n)^1^	19,484	1,005	1,077	649	19,284	4,139	4,267	3,681
Obtained overall imputation accuracy (n)	672,416	690,895	690,823	691,251	252,796	267,941	267,813	268,399
average overall imputation accuracy (r)	0.70	0.83	0.88	0.89	0.59	0.74	0.80	0.82
standard deviation of r	0.32	0.27	0.25	0.24	0.37	0.32	0.29	0.28

^1^No variation was present in the genotype dosages of at least one of the 5 corresponding cross-validation sets, therefore the imputation accuracy (correlation) could not be computed.

^2^In scenario HOL20 the reference sets were the same as the validation sets, therefore all variants without variation in at least one cross-validation reference set are the same as the variants without variation in observed genotypes of the validation sets.

Although the 777 K SNP chip contained only SNP, the sequence variants contained both SNP and indels [12,19], and both were imputed. Results showed that on average the imputation accuracy for indels was approximately 0.12 lower than for SNP in all four scenarios and on both chromosomes (Table 2).

**Table 2:** **Average imputation accuracy** (**r**) **of SNP and short insertions and deletions** (**indels**) **on chromosome 1 and 29**

	**Chromosome 1**				**Chromosome 29**			
	**SNP (1,069,830)**		**Indels (76,351)**		**SNP (405,507)**		**Indels (26,210)**	
**Scenario**	**n**	**r**	**n**	**r**	**n**	**r**	**n**	**r**
HOL20	630,092	0.71	42,324	0.56	238,473	0.60	14,323	0.47
MIX80	646,872	0.84	44,023	0.71	252,571	0.75	15,370	0.64
HOL80	646,796	0.88	44,027	0.76	252,439	0.81	15,374	0.70
MIX140	647,200	0.89	44,051	0.78	252,996	0.83	15,403	0.73

### Multi-breed scenarios benefitted from persistency of phase across breeds

**Figure 3:**
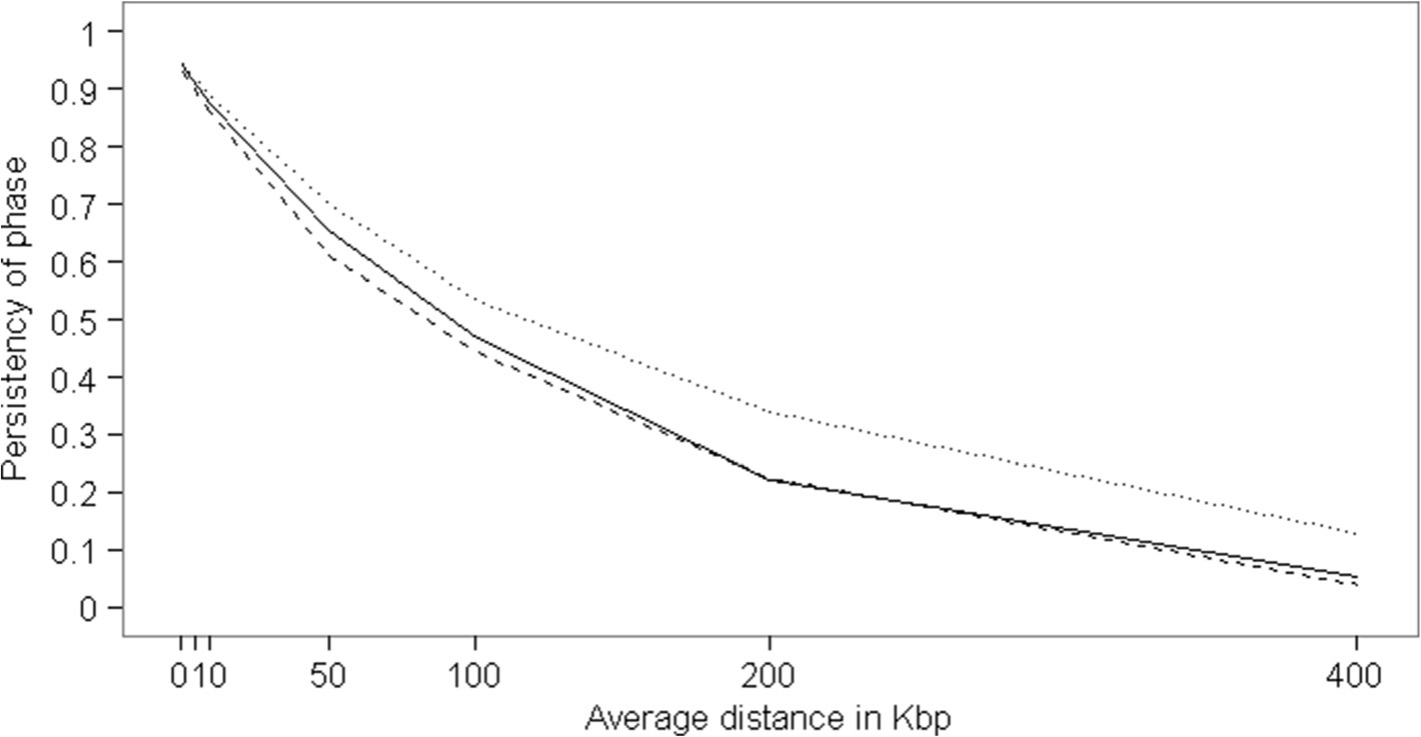
**Persistency of phase across breeds.** Persistency of phase between Holstein and Brown Swiss (solid line), Holstein and Jersey (dashed line), Holstein and Nordic Red Dairy Cattle (dotted line) on chromosome 1.

When the other breeds were included in the reference population in MIX80 the imputation accuracy increased compared to HOL20, and the same was true for the MIX140 scenario compared to HOL80. This indicates that BSW, JER and RDC had haplotypes in common with Holstein. On chromosome 1, the distance between consecutive SNP on the 777 K SNP chip was on average 3,781 bp. The persistency of phase between Holstein and the three other breeds ranged between 0.89 and 0.95 at a distance of 0 to 6 kb on chromosome 1 (Figure 3). This high persistency of phase of variants at a distance similar to the average distance between SNP on the 777 K SNP chip confirms that at this distance the other breeds were valuable for imputation. However, the persistency of phase declined strongly with increasing distance between variants (Figure 3). The maximum distance between SNP on the 777 K SNP chip on chromosome 1 was 162 kb, and at this distance the persistency of phase was approximately 0.3. Therefore, at such distance the other breeds in MIX80 might have contributed little to the imputation accuracy of Holsteins, albeit such large distances between SNP on the 777 K SNP chip were exceptional (i.e. on chromosome 1 the 90% quantile was 7,880 bp).

### Variants with low MAF

In general, variants with very low MAF did not obtain an overall (over all 5 cross-validations) imputation accuracy, because either the observed genotypes or the imputed genotypes, or both, were monomorphic in at least one of the five cross-validations. About 58 to 63% of the variants to impute obtained an overall imputation accuracy (Table 1). Therefore, imputation accuracy of variants with low MAF were investigated per cross-validation. Results from one cross-validation on chromosome 1 are shown, but are similar for the other cross-validations on chromosome 1.

Table 3 shows the average imputation accuracy and average MAF of scenarios HOL80 and MIX80 categorized by MAF in the HOL80 reference population for this single cross-validation on chromosome 1. The average imputation accuracy of scenarios MIX80 and HOL80 for variants with a MAF higher than 0.1 in the HOL80 reference population was fairly similar and most variants were imputed with high accuracy (r_MIX80_ = 0.90, r_HOL80_ = 0.92). With a MAF ranging from 0.1 to 0.01875 in the HOL80 reference population the average imputation accuracy reduced considerably in both scenarios (Table 3). With a MAF in the HOL80 reference population of 0.0125 or lower the average imputation accuracy dropped even further, but for such variants MIX80 performed on average better than HOL80 (Table 3). With such low MAF in the HOL80 reference population MIX80 performed better due to a higher MAF in the reference population, since the minor allele was often segregating in at least one of the other breeds. Comparing HOL80 with MIX140 showed even a stronger benefit for the MIX140 scenario, especially for variants with a MAF smaller than 0.03 (Table 4).

**Table 3:** **Average imputation accuracy for scenarios HOL80 and MIX80 per category of minor allele frequency**

**MAF range**	**n alleles** ^**1**^	**n variants**	**HOL80**		**MIX80**	
			**MAF**	**r**	**MAF**	**r**
> 0.1	> 16	525,575	0.27237	0.92	0.26094	0.90
0.05625-0.1	9-16	141,708	0.07655	0.78	0.09741	0.71
0.03125-0.05	5-8	89,926	0.04076	0.67	0.06922	0.57
0.01875-0.025	3-4	51,491	0.02193	0.57	0.05642	0.51
0.0125	2	22,135	0.01250	0.44	0.05434	0.51
0.00625	1	18,384	0.00625	0.20	0.05014	0.49

^1^number of minor alleles present in the HOL80 reference population at corresponding MAF range in the HOL80 reference population.

Average imputation accuracy (r) and average minor allele frequency (MAF) of the reference population for scenarios HOL80 and MIX80 for variants on chromosome 1 per category of MAF range in the HOL80 reference population. Results are only shown for one cross-validation, but are similar for all cross-validations on chromosome 1.

**Table 4:** **Average imputation accuracy for scenarios HOL80 and MIX140 per category of minor allele frequency**

**MAF range**	**n alleles** ^**1**^	**n variants**	**HOL80**		**MIX140**	
			**MAF**	**r**	**MAF**	**r**
> 0.1	> 16	525,647	0.27237	0.92	0.27159	0.93
0.05625-0.1	9-16	143,313	0.07639	0.78	0.08784	0.79
0.03125-0.05	5-8	94,021	0.04073	0.66	0.05596	0.69
0.01875-0.025	3-4	56,235	0.02187	0.56	0.03884	0.62
0.0125	2	25,698	0.01250	0.42	0.03216	0.56
0.00625	1	25,101	0.00625	0.19	0.02385	0.45

^1^number of minor alleles present in the HOL80 reference population at corresponding MAF range in the HOL80 reference population.

Average imputation accuracy (r) and average minor allele frequency (MAF) of the reference population for scenarios HOL80 and MIX140 for variants on chromosome 1 per category of MAF range in the HOL80 reference population. Results are only shown for one cross-validation, but are similar for all cross-validations on chromosome 1.

### Holstein specific variants

On chromosome 1 there were 182,964 Holstein specific variants to be imputed, and 60,202 on chromosome 29. Here, Holstein specific variants were defined as variants that were segregating in the 100 Holstein but not in any of the individuals of the other three breeds used in this study. The Holstein specific variants showed differences in imputation accuracy between the scenarios. On chromosome 1, the average (overall) imputation accuracy for Holstein specific variants was 0.42 (n = 34,071) for HOL20, 0.52 (n = 35,478) for MIX80, 0.79 (n = 35,476) for HOL80, and 0.80 (n = 35,484) for MIX140. On chromosome 29, the average (overall) imputation accuracy for Holstein specific variants was 0.32 (n = 10,483) for HOL20, 0.41 (n = 11,181) for MIX80, 0.69 (n = 11,185) for HOL80, and 0.71 (n = 11,208) for MIX140. So when other breeds were added (MIX80) to the small reference population (HOL20) the average imputation accuracy increased with approximately 0.10 on both chromosomes, even though the SNP were not segregating in the other breeds. Obviously, when more individuals from the same breed were added (HOL80) this increase was a lot larger (0.28-0.37), but adding the other breeds to the reference population of 80 Holstein (MIX140) improved the imputation accuracy of Holstein specific variants only marginally (0.01-0.02).

As explained above, variants with very low MAF did not obtain an overall imputation accuracy. Of the Holstein specific variants only 17 to 19% obtained an overall imputation accuracy. Therefore, results of an individual cross-validation set on chromosome 1 are shown in the next section to gain more insight in imputation accuracy of Holstein specific variants with low MAF.

Table 5 shows the average imputation accuracy and average MAF of Holstein specific variants for scenarios HOL80 and MIX80, categorized by MAF in the HOL80 reference population. For Holstein specific variants with low MAF scenario HOL80 resulted in general in higher imputation accuracies as compared to scenario MIX80. For Holstein specific variants the imputation accuracy of MIX80 depended on the frequency of the minor allele in the 20 Holsteins present in MIX80. On average the MIX80 scenario obtained reasonable accuracies (r_MIX80_ = 0.78) for Holstein specific variants when the MAF of the variants was 0.1 or higher in Holstein (Table 5), but with lower MAF chances were higher that the minor allele was underrepresented in those 20 Holstein. Accordingly, Table 5 shows that the imputation accuracy (and MAF) of the MIX80 scenario dropped much faster with decreasing MAF of the Holstein specific variants as compared to the HOL80 scenario.

**Table 5:** **Average imputation accuracy of Holstein specific variants for HOL80 and MIX80 per category of MAF**

**MAF range**	**n alleles** ^**1**^	**n variants**	**HOL80**		**MIX80**	
			**MAF**	**r**	**MAF**	**r**
> 0.1	> 16	15,736	0.16529	0.87	0.04369	0.78
0.05625-0.1	9-16	23,057	0.07367	0.79	0.02289	0.63
0.03125-0.05	5-8	14,825	0.04061	0.63	0.01046	0.30
0.01875-0.025	3-4	9,185	0.02152	0.54	0.00855	0.24
0.0125	2	2,616	0.01250	0.38	0.00679	0.13
0.00625	1	1,663	0.00625	0.18	0.00625	0.14

^1^number of minor alleles present in the HOL80 reference population at corresponding MAF range in the HOL80 reference population.

Average imputation accuracy (r) and average minor allele frequency (MAF) of the reference population for scenarios HOL80 and MIX80 for Holstein specific variants on chromosome 1 per category of MAF range in the HOL80 reference population. Results are only shown for one cross-validation, but are similar for all cross-validations on chromosome 1.

When the MAF in HOL80 was 0.0125 or higher the MIX80 scenario outperformed the HOL20 scenario for Holstein specific variants (results not shown). Similarly, MIX140 outperformed HOL80 marginally for Holstein specific variants (Table 6). This suggests that in general the MIX scenarios benefitted from 60 additional animals, even though they did not carry the minor allele. With all reference populations, imputation of Holstein specific variants was poor when the MAF was extremely low (Tables 5 and 6).

**Table 6:** **Average imputation accuracy of Holstein specific variants for HOL80 and MIX140 per category of MAF**

**MAF range**	**n alleles** ^**1**^	**n variants**	**HOL80**		**MIX140**	
			**MAF**	**r**	**MAF**	**r**
> 0.1	> 16	15,741	0.16528	0.87	0.09452	0.89
0.05625-0.1	9-16	24,641	0.07293	0.80	0.04167	0.81
0.03125-0.05	5-8	18,872	0.04046	0.63	0.02312	0.64
0.01875-0.025	3-4	13,865	0.02143	0.51	0.01224	0.54
0.0125	2	6,139	0.01250	0.33	0.00714	0.34
0.00625	1	8,266	0.00625	0.15	0.00357	0.15

^1^number of minor alleles present in the HOL80 reference population at corresponding MAF range in the HOL80 reference population.

Average imputation accuracy (r) and average minor allele frequency (MAF) of the reference population for scenarios HOL80 and MIX140 for Holstein specific variants on chromosome 1 per category of MAF range in the HOL80 reference population. Results are only shown for one cross-validation, but are similar for all cross-validations on chromosome 1.

## Discussion

The aim of this study was to determine the consequences of splitting sequencing effort over multiple breeds for imputation accuracy from high-density SNP panels towards whole-genome sequence. Although a larger sequenced reference population from the same breed is preferred, this paper shows that addition of sequenced individuals from other breeds to reference populations of limited size (i.e. MIX80 versus HOL20) also increased the imputation accuracy. Especially variants with low MAF in Holstein that were also segregating in the other breeds benefitted from multi-breed reference populations, while Holstein specific variants benefitted from the larger Holstein reference population. In any case, imputation with a reference population of 80 animals (single or multi-breed) performed better than only 20 animals in a single-breed reference population. Thus, when sequencing effort is limiting and interest lays in multiple breeds or lines, splitting the effort over a number of breeds and combining the reference populations provides a good alternative that allows imputation of each breed.

Two chromosomes were analysed and showed differences in imputation accuracy. The average imputation accuracy on chromosome 29 was lower than the average imputation accuracy on chromosome 1. This might be due to the limited number of SNP on the 777 K SNP chip in certain regions on chromosome 29 as shown by Daetwyler et al. [12], indicating not only the density but also the distribution of SNP on chips is important. Besides such gaps there could also be mapping errors complicating the imputation process as indicated by Erbe et al. [1], underlining the need for an improved reference genome. This indicates that even though genome-wide average imputation accuracy might be high, there remain poor imputed regions.

### Multi-breed imputation to whole-genome sequence

De Roos et al. [18], suggested that in cattle 300 K markers would be sufficient for QTL mapping and genomic prediction across breeds, with 300 K markers the distance between marker and QTL would be ~5 kb. For the dairy breeds studied here, the persistency of phase at a distance of 5 kb on chromosome 1 was > 0.88, while the average distance between SNP on the 777 K SNP chip was only 3,781 bp on chromosome 1. Even at 10 kb the persistency of phase on chromosome 1 was still above 0.85, however at 50 kb the persistency of phase decreased to 0.61 between Holstein and Jersey. Here, multi-breed imputation to whole-genome sequence seemed to similarly benefit from the persistency of phase between the breeds with the high marker density. However, for single-breed imputation to whole-genome sequence a similar marker density is required to obtain reasonable imputation accuracy, as Van Binsbergen et al. [5] showed that imputation accuracy from 50 K SNP to sequence within Holstein was 0.46, while imputation from 777 K SNP to sequence was 0.83 in their study both with a reference population of 90 Holstein. In current study, imputation from 777 K to sequence resulted in an imputation accuracy of 0.70 on chromosome 1 and of 0.59 on chromosome 29 with as little as 20 Holsteins in the reference population, suggesting that the marker density is more important than the size of the reference population.

In this study, a very small initial reference population was used to compare to a multi-breed reference population (HOL20 versus MIX80). This is typical for current sequenced populations, but might not be representative for future situations as the number of sequenced individuals will accumulate over time or be shared in projects like the 1,000 Bull Genome project. Imputation studies analysing imputation from low or medium-density SNP chips to high-density SNP chips showed that adding other breeds has little impact on imputation accuracy when the within breed reference populations is already large [6,11]. In agreement with current study, Daetwyler et al. [12] and Brøndum et al. [14] recently showed that adding individuals from other breeds is beneficial when within-breed reference populations are numerically small, e.g. around 15, 40 or 50 sequenced reference bulls, but imputation accuracy improved only marginally by adding sequenced animals from other breeds to a sequenced reference population of 95 to 131 Holsteins. From present study it can be concluded that, even though the average imputation accuracy improved only marginally when other breeds were included (MIX140 versus HOL80), the benefit of including additional breeds in a relatively large reference population was the increase in imputation accuracies for variants with low MAF that were segregating in the other breeds. In addition, IMPUTE2 [20] yields higher imputation accuracies for low MAF variants compared to BEAGLE for imputation up to sequence using multi-breed reference populations [14], thus IMPUTE2 in combination with multi-breed reference populations gives currently the best results for imputation of low MAF variants.

So with sufficient persistency of phase between breeds a multi-breed reference population can be of great value for imputation when within-breed reference populations are smaller than roughly 80 animals, and remain of value for low MAF variants segregating in the other breeds for reference populations of 80 individuals or more. Whether the results of our study can be translated to other breeds and species depends strongly on the persistency of phase between the breeds of interest, but can be compensated by the density of markers. Even when high density chips are not available (e.g. in pigs), the sequenced reference set can function as a high density reference set for imputation in two (or more) steps by masking a large part of the sequence to mimic a high density chip.

### Lower imputation accuracy for indels

For all four scenarios the imputation accuracy of indels was lower than for SNP. This might be because SNP and indels arise due to different mechanisms in DNA replication or repair and differ in mutation rate, which can lead to differences in allele frequency and thus in differences in LD between SNP and indels. However, in humans there appeared to be useful LD between short indels and SNP on commercially available genotyping chips [21]. For the average SNP distance on chromosome 1 with the 777 K chip (3,781 bp) there was useful LD (${r}_{LD}^2=0.25$ at 0-1 kb, ${r}_{LD}^2=0.19$ at 5-6 kb) between typed chip-SNP and indels in the sequence data. However, the LD between typed chip-SNP and SNP in the sequence data is larger (${r}_{LD}^2=0.40$ at 0-1 kb, ${r}_{LD}^2=0.29$ at 5-6 kb) and stays useful over a longer distance between the SNP (${r}_{LD}^2>0.2$ at 10 kb). Therefore, the difference in LD between SNP and indels and the SNP on the chip could be the reason that the imputation accuracy of SNP was on average 0.12 higher than for indels.

### Breed specific variants

For Holstein specific variants the HOL80 scenario performed much better than MIX80, but the MIX80 scenario outperformed the HOL20 scenario, and likewise the MIX140 outperformed HOL80. The minor allele count in HOL20 and MIX80 is equal, since the MIX80 scenario contained exactly the same 20 Holstein individuals as the HOL20 scenario, but the MAF is lower in MIX80 due to the additional 60 individuals from the other breeds that did not carry the minor allele, the same is true for HOL80 and MIX140. Still MIX80 and MIX140 had higher imputation accuracy as compared to HOL20 and HOL80, respectively, therefore, this suggests that the haplotypes of the other breeds excluded some haplotypes present in the validation set from harbouring the minor allele. The fact that Holstein specific variants were in general poorly imputed might be of concern if interest lays in breed specific characteristics. In such cases, a large single-breed reference population would be preferred.

## Conclusions

The aim of this study was to determine the consequences of splitting whole-genome sequencing effort over multiple breeds for imputation accuracy. With a base reference population of 20 Holstein individuals imputation accuracy on chromosome1 is poor (r = 0.70), adding 60 individuals from other dairy breeds improved the imputation accuracy considerably (r = 0.83), however when the same amount of animals from the Holstein breed were added the accuracy improved to 0.88, while adding the 3 other breeds to the reference population of 80 Holstein improved the average imputation accuracy marginally to 0.89. For chromosome 29, the average imputation accuracy was lower. Especially variants with low MAF in Holstein that were also segregating in the other breeds benefitted from the multi-breed reference population, while Holstein specific variants benefitted from the larger Holstein reference population. In any case, imputation with a reference population of 80 animals (single or multi-breed) performed better than only 20 animals in a single-breed reference population. When sequencing effort is limiting and interest lays in multiple breeds or lines, splitting the effort over a number of breeds and combining the reference populations provides a good alternative that allows imputation of each breed.
